# Risk prediction model for knee pain in the Nottingham community: a Bayesian modelling approach

**DOI:** 10.1186/s13075-017-1272-6

**Published:** 2017-03-20

**Authors:** G. S. Fernandes, A. Bhattacharya, D. F. McWilliams, S. L. Ingham, M. Doherty, W. Zhang

**Affiliations:** 1 Division of Rheumatology, Orthopaedics and Dermatology, School of Medicine, University of Nottingham, Nottingham, UK; 2 Arthritis Research UK Centre for Sport, Exercise and Osteoarthritis, Nottingham, UK; 3 Arthritis Research UK Pain Centre, University of Nottingham, Nottingham, UK; 4 Academic Rheumatology, School of Medicine, Nottingham University, Nottingham City Hospital, Nottingham, NG5 1PB UK; 5 Faculty of Life Sciences, University of Manchester, Carys Bannister Building, Dover Street, Manchester, M13 9PL UK

**Keywords:** Knee pain, Bayesian statistics, Prediction modelling, Musculoskeletal epidemiology

## Abstract

**Background:**

Twenty-five percent of the British population over the age of 50 years experiences knee pain. Knee pain can limit physical ability and cause distress and bears significant socioeconomic costs. The objectives of this study were to develop and validate the first risk prediction model for incident knee pain in the Nottingham community and validate this internally within the Nottingham cohort and externally within the Osteoarthritis Initiative (OAI) cohort.

**Methods:**

A total of 1822 participants from the Nottingham community who were at risk for knee pain were followed for 12 years. Of this cohort, two-thirds (*n* = 1203) were used to develop the risk prediction model, and one-third (*n* = 619) were used to validate the model. Incident knee pain was defined as pain on most days for at least 1 month in the past 12 months. Predictors were age, sex, body mass index, pain elsewhere, prior knee injury and knee alignment. A Bayesian logistic regression model was used to determine the probability of an OR >1. The Hosmer-Lemeshow χ^2^ statistic (HLS) was used for calibration, and ROC curve analysis was used for discrimination. The OAI cohort from the United States was also used to examine the performance of the model.

**Results:**

A risk prediction model for knee pain incidence was developed using a Bayesian approach. The model had good calibration, with an HLS of 7.17 (*p* = 0.52) and moderate discriminative ability (ROC 0.70) in the community. Individual scenarios are given using the model. However, the model had poor calibration (HLS 5866.28, *p* < 0.01) and poor discriminative ability (ROC 0.54) in the OAI cohort.

**Conclusions:**

To our knowledge, this is the first risk prediction model for knee pain, regardless of underlying structural changes of knee osteoarthritis, in the community using a Bayesian modelling approach. The model appears to work well in a community-based population but not in individuals with a higher risk for knee osteoarthritis, and it may provide a convenient tool for use in primary care to predict the risk of knee pain in the general population.

**Electronic supplementary material:**

The online version of this article (doi:10.1186/s13075-017-1272-6) contains supplementary material, which is available to authorized users.

## Background

People of all ages can experience persistent knee pain, and one-fourth of the population over the age of 50 years in the United Kingdom is affected [1, 2]. Knee pain can limit lower limb function, induce disability and distress, and reduce quality of life, resulting in high societal and health-economic costs [3]. Knee pain commonly associates with knee osteoarthritis (KOA) in middle-aged and older people and is the main reason why 20% of people with KOA give up work or retire earlier by 8 years [4]. This burden is increasing as a result of ageing populations, increasing prevalence of obesity and lack of effective preventive strategies.

However, the association between knee pain and KOA continues to be debated. One reason for this is the common discordance between radiographic KOA and knee pain [5]. Self-reported knee pain can occur both with and without any radiographic osteoarthritis (OA) change, and such discrepancies could be due to x-ray views used, definition of pain, OA grading scores and population characteristics studied. Regardless of the debate, what is clear is knee pain is a common malady [6], KOA is one of many risk factors associated with this malady, and it is the knee pain that causes a patient to consult.

In radiographic OA, the Kellgren and Lawrence (KL) composite score is often used to classify the disease which comprises the presence of osteophytes predominantly and, to an extent, joint space narrowing. The prevalence of radiographic KOA using the KL score in adults over the age of 45 years varies from 19% to 37% [5]. The prevalence of self-reported knee pain was 35% in men and 62% of women over the age of 40 years [7]. In the National Health and Nutrition Examination Survey I study, of 6880 participants, 14.6% reported knee pain, and only 15% of these had KL scores demonstrating structural OA changes [8]. In 1992, Hadler remarked, ‘The epidemiology of osteoarthritis and the epidemiology of pain have little in common, not nothing in common, but surprisingly little’ ([9]; pg 598). This distinction is important because OA management guidelines, healthcare spending, and a healthcare practitioner’s diagnosis, treatment and management are targeted at reducing pain and associated symptoms as opposed to treating structural radiographic changes. It is knee pain and associated symptoms in KOA that lead to consultations as well as social and economic burdens [10–12]. Importantly, from a patient’s perspective, it is the knee pain that limits everyday activities such as getting out of bed in the morning or climbing stairs. An understanding of the risk factors that contribute to and predict incident knee pain and knee pain progression instead of focussing on structural KOA is arguably a more insightful and useful clinical tool.

The first risk prediction model for incidence and progression of KOA was developed by Zhang and colleagues [6] on the basis of a 12-year retrospective community cohort (Nottingham) using conventional risk factors such as age, sex, body mass index (BMI), family history of OA, occupational risk and joint injury. The study reported that reducing obesity would have an effect on patient outcomes and radiographic KOA development. Another prognostic prediction model for incident KOA was developed in a larger cohort (Rotterdam Study II and Chingford) [13] using clinical, genetic and biochemical risk factors which showed a moderate predictive value for incident KOA based on genetics.

There have been no risk prediction models developed for incident or progressive knee pain, and because knee pain and KOA present distinctly in a clinical setting, further investigation into whether known and unknown risk factors affect knee pain outcomes is the purpose of the present study. We sought to develop the first knee pain risk prediction model, regardless of any underlying structural changes of KOA, to provide a convenient tool for use in primary care to predict the risk of this common malady. As a result, conventional risk factors that can be measured easily in a primary care setting were included, such as age, sex, BMI, self-reported varus and valgus alignment, and joint injury [14]. The objectives of this study were (a) to develop a risk prediction model for incident knee pain in community participants in Nottingham, UK; and (b) to validate this internally within the Nottingham community and externally with the Osteoarthritis Initiative (OAI) cohort from the United States.

## Methods

### Study design and setting

A 12-year retrospective cohort study was undertaken involving four general practices in North Nottinghamshire, UK. The study was approved by the Nottinghamshire County Primary Care Trust, Nottingham University Hospitals NHS Trust (reference 07RH004), and by the Nottingham Research Ethics Committee 1 (reference 07/H0403/111).

### Participants

**Fig. 1:**
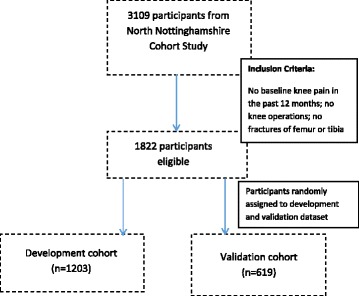
Derivation of the Nottingham study cohorts

Individuals were recruited from two baseline community postal questionnaire studies for knee pain [14]. Baseline data were collected between 1996 and 1999 from 9429 adults aged 40–79 years on the general practice registers. A follow-up survey was undertaken during 2007–2008 with 5479 individuals who were still registered with the general practices and eligible for the study. People with terminal illness, psychiatric illness and severe dementia were excluded [14]. Of 3109 people followed for 12 years, 1822 were at risk for knee pain (i.e., reported no knee pain in either knee in the year prior to baseline), and these individuals formed the cohort for the present study. We randomly selected two-thirds of the cohort (*n* = 1203) to develop the model and the remaining one-third (*n* = 619) to validate the model (Fig. 1). In addition, the model performance was examined in an external cohort with high risk for KOA: the U.S. OAI cohort study. The OAI was selected because it is a publicly available dataset which had baseline data with an adequate follow-up period (8 years) as well as data on the known risk factors or predictors of knee pain. In the OAI cohort study, 855 participants with complete datasets had no knee pain in either knee at baseline, and these participants were followed for 8 years for incident knee pain. For incident knee pain in OAI, the question ‘Have you had pain on most days of at least 1 month in the past 12 months’? was used to determine pain status at baseline and then at follow-up 8 years later. Other parameters included were age, sex, BMI, presence of a knee injury (‘Have you ever suffered a significant injury to either of your knees’?) and pain elsewhere.

### Definition of incident knee pain

The definition of knee pain in this study was the presence of self-reported knee pain in and around a knee on most days for at least 1 month. People with incident knee pain were those with no knee pain for the past 12 months at baseline and who reported knee pain in the follow-up questionnaire. We also excluded those who reported knee operations or long-bone leg fractures (femur or tibia) at baseline as well as during the follow-up.

### Knee pain prediction models

#### Logistic regression model

Logistic regression modelling was used for incident knee pain prediction (Eq. 1 below), where *p* is the probability of knee pain, α is the constant and β_i_ is the logarithm value of OR for a specific predictor *X*_i_. For convenient prediction, we kept age and BMI as continuous variables and other risk factors as dichotomous or categorical variables. When a binary outcome variable (knee pain) is modelled using logistic regression, the logit transformation of the outcome can be assumed to have a linear relationship with predictor values. Therefore, the logit operator maintains the linearity of the model and allows the calculation of a probability of knee pain (*p*), given the different sets of predictors (e.g., *X*_i_, *X*_ii_) [6, 15].

1 $$ \mathrm{Logit}= \ln \left(\mathrm{p}/1\ \hbox{-}\ \mathrm{p}\right)=\upalpha +\upbeta 1\mathrm{Xi}+\upbeta \mathrm{iXii}+\dots $$

#### Bayesian techniques

A logistic regression model was deployed using Bayesian inference. Posterior distribution of the parameters in the model was simulated using data and assumed prior distribution on the parameters. This approach provides flexibility of calculating certain types of posterior probabilities to enhance the interpretation from the model; for example, *p*(OR >1 data) for all the risk factors, which can be better interpreted than having a *p* value and making a decision based on it. This type of probability provides more information about the role of the corresponding predictor in the model. Non-informative prior distributions were selected for associated risk parameters. Normal distributions were used as prior distributions for all risk parameters, with the most common choice of prior mean being zero and prior SD being 100 to make it non-informative. All the study results were analysed using STATA SE 13 software (StataCorp, College Station, TX, USA), apart from Bayesian inference, which was analysed using SAS version 9.43 software (PROC MCMC; SAS Institute, Cary, NC, USA).

#### Predictors

The predictive risk factors associated with knee pain were drawn from the literature and included the well-established constitutional predictors: age (in years); sex (0 = male, 1 = female); family history of OA (family history of joint replacement and nodes, 0 = no, 1 = yes); index/ring finger ratio (second digit/fourth digit [2D:4D] ratio; 0 = patterns 1 and 2, 1 = pattern 3) using a validated line drawing in the questionnaire [16]; biomechanical risk factors such as baseline BMI (in kilograms per metre squared); presence of significant previous knee injury (0 = no, 1 = yes); pain elsewhere (pain in two specific regions [hip and back], 1 = yes or 0 = no pain); self-reported baseline varus knee alignment (1 = yes, 0 = no) or self-reported baseline valgus knee alignment (1 = yes, 0 = no) using a validated line drawing [17]; back pain ever (0 = no, 1 = yes); knee pain ever (0 = no, 1 = yes); presence of any finger nodes (0 = no, 1 = yes); psychological risk factors from the 36-item Short Form Health Survey, such as mood or mental health component scores (tertiles with increasing order representing lower score); and general health (quartiles with increasing order representing lower scores). Data on analgesic use were not included in our model. All predictors for the Nottingham cohort were taken from baseline. If the predictors were significant, these were extracted from the OAI database at their baseline time point. The exception to the predictor description was knee alignment in the OAI cohort because this was a baseline measurement assessed using a goniometer to determine whether alignment was neutral, varus or valgus as opposed to the validated line drawing. All predictors were chosen at a person- rather than a knee-specific level because the risk factors for knee pain would differ not on the basis of laterality, but rather the absolute presence of symptoms or not.

### Validation

#### Calibration and discrimination

Calibration and discrimination were examined for the model performance. Calibration assesses how closely the predicted probabilities reflect actual risk. A risk score was calculated for each individual using Eq. 1. The higher the risk score, the greater the risk of knee pain. The individuals were classified into different groups (deciles) according to the risk scores. Observed and predicted frequencies of the disease in subgroups were calculated. The Hosmer-Lemeshow χ^2^ statistic (HLS) for goodness of fit was used for calibration to compare observed and predicted risk deciles whereby small values indicated good calibration [18]. Discrimination examines the ability to correctly classify subjects into different groups. To assess this parameter, the AUC was used. The ROC presents a curve of sensitivity (*y*-axis) against 1 − specificity (*x*-axis) at different cut-off points of the risk score. Larger values of the ROC indicate better discriminative power [6]. Case scenarios were given to examine the model performance in individual cases. In addition, both calibration and discrimination tests were used to examine the performance of the model in OAI. Only those participants with full reports (all predictors) were selected for the models, and incomplete data was treated as missing.

## Results

### Population characteristics

The Nottingham cohort had a mean age at baseline of 56 years (SD ±8.84) and a mean BMI of 25.13 kg/m^2^ (SD ±3.4), and 55.38% were women. The OAI sample with no knee pain at baseline (*n* = 855) had a mean age of 61.30 years (SD ±8.98) and a mean BMI of 27.46 (SD ±4.69), and 59.32% were women. Of 1822 participants at risk in the Nottingham cohort, 533 (29%) developed knee pain in 12 years. Of 855 participants at risk in OAI, 333 (47%) developed knee pain in 8 years. Further details of these two cohorts are shown in Table 1.

**Table 1:** Characteristics of the study populations at baseline

	Nottingham	OAI	*p* Value
Setting	Community	Hospital	
Number of participants	1822	855	
Age^a^, years (mean ± SD)	56.01 ± 8.84	63.47 ± 9.41	<0.01
BMI^a^, kg/m^2^ (mean, SD)	25.13 ± 3.40	27.46 ± 4.69	<0.01
Women (*n*)	1009 (55.38%)	420 (59.32%)	0.22
Knee pain at follow-up	533 (29.66%)	333 (47.03%)	<0.01
Pain elsewhere (%)	968 (54.05%)	370 (52.26%)	<0.01
Knee injury (%)	201 (11.19%)	266 (37.57%)	<0.01
Varus knee alignment (%)	60 (3.37%)	212 (24.8%)	<0.01
Valgus knee alignment (%)	44 (2.47%)	384 (44.9%)	<0.01

*BMI* Body mass index, *OAI* Osteoarthritis Initiative

^a^ Statistical difference assessed using *t* test for continuous variables. All other variables (categorical) assessed using χ^2^ test

### Risk prediction model

The development data were used to determine which parameters were of significance (*p* < 0.05), and these were identified as age, sex, BMI, knee injury, pain elsewhere and presence of a varus knee or valgus knee. The following parameters were not significant: family history of OA, 2D:4D finger ratio, presence of finger nodes and psychological risk factors (*p* > 0.05). Table 2 presents the ORs for each parameter and, using Bayesian analysis, determines the posterior probability of the OR being >1.

**Table 2:** ORs and 95% CIs for individual risk factors determined in development sub-group

Parameter	OR	95% CI	Posterior probability of OR >1
Age	1.01	1.00–1.03	0.84
Sex	1.33	0.99–1.72	0.92
BMI	1.11	1.07–1.15	1.00
Knee injury	4.91	3.27–7.22	1.00
Pain elsewhere	2.49	1.83–3.30	1.00
Knee alignment	3.93	2.14–6.57	1.00

*BMI* Body mass index

The following risk prediction model for knee pain incidence was then developed using the development dataset:

$$ Logit = \mathit{\hbox{-}} 4.39+ 0.0095\; age+ 0.21\; female+ 0.06\; body\; mass\; index+ 1. 51\; knee\; injury+ 1. 03\; pain\; elsewhere+ 1. 30\; varus\; knee+ 0.946\; valgus\; knee\;\left( model\; 1\right) $$

Therefore, we used the following formula to calculate the likelihood of developing incident knee pain:

$$ \mathrm{Percentage}\;\mathrm{likelihood}\;\mathrm{of}\;\mathrm{knee}\ \mathrm{pain}:\left(1/\left(1+{\mathrm{e}}^{-\mathrm{logit}}\right)\right)\times 100 $$

Using this model and the subsequent formula of percentage likelihood in hypothetical case scenarios, a woman aged 65 years with a BMI of 32 kg/m^2^, a history of prior knee injury, no pain elsewhere, and varus knee alignment is 76.3% likely to develop knee pain at 12-year follow-up. Similarly, if we were to take the case of a 50-year-old man with a BMI of 26 kg/m^2^, no history of knee injury or pain elsewhere, and a neutral knee alignment, he would be 12.61% likely to develop knee pain at follow-up.

### Validation

#### Calibration

**Fig. 2:**
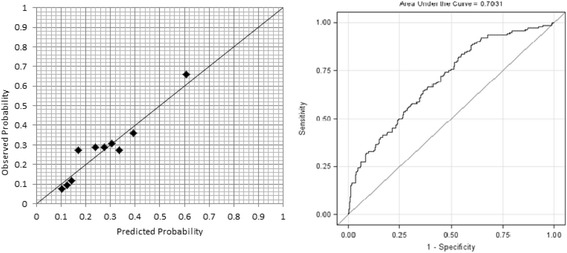
Calibration and discrimination in the Nottingham community

In the validation dataset (*n* = 619), the HLS for goodness of fit was 7.17 (*p* = 0.52). This indicates that the logit model is explaining the validation data well. Figure 2 demonstrates the observed and predicted probabilities when plotted per decile of the validation data using the HLS.

#### Discrimination

The AUC for the internal cohort showed a moderate discriminative ability of model 1 (ROC 0.70, 95% CI 0.65–0.75) with a sensitivity of 93.5% and specificity of 31.5%. This is also represented in Fig. 2.

#### Model performance in OAI

**Fig. 3:**
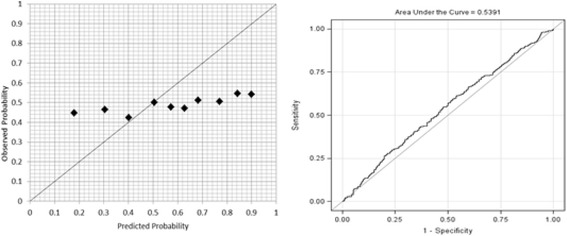
Calibration and discrimination in the Osteoarthritis Initiative population

When tested model 1 in the OAI dataset (*n* = 855), the HLS was poor (5866.28, *p* < 0.01). This indicates that the logit model does not explain the OAI data very well. The AUC for the OAI cohort also showed a poor discriminative ability of model 1 (ROC 0.54, 95% CI 0.50–0.58), with a sensitivity of 73% and specificity of 31%. These are demonstrated in Fig. 3 for the observed and predicted probabilities when plotted per decile of the OAI data using the HLS.

## Discussion

To our knowledge, this is the first risk prediction model for knee pain in a general population sample. Conventional risk factors that can be measured easily in primary care were included in this model to increase its utility, including age, sex, BMI, history of knee injury, pain elsewhere and knee alignment. The following are the main findings:

1. Knee pain can be predicted by conventional risk factors.
2. The likelihood of this prediction (calibration) is better in the general population than in individuals with high risk of KOA.
3. The discrimination is also better in the general population than in the high-risk population (OAI).
4. The model has high sensitivity (95%) but lower specificity (32%), so it is more useful for screening possible knee pain cases rather than for confirming the diagnosis.

This is also the first prediction model to use Bayesian inference technique. This has at least two advantages: (1) It usually gives more precise estimates (i.e., narrower CIs) of the risk prediction [19], and (2) it provides a posterior probability of having OR >1 rather than a *p* value. The latter gives a degree of likelihood that a person would have the disease, given an exposure to the risk factor(s), not just a false-positive error from a statistical test. This is an advantage of the Bayesian over the frequentist statistics, where uncertainty is measured by the probability of having a disease, not the probability of making a false-positive error [20].

Knee injury, presence of pain elsewhere and varus knee alignment were the strongest clinical predictors of knee pain using our model. Not surprisingly, the strongest predictor was knee injury, which is a well-known local biomechanical risk factor for subsequent development of KOA, of which knee pain is a major symptom [2]. The precise relationships between joint injury and development of post-traumatic OA and pain are poorly understood. However, any major insults to the articular cartilage, menisci and ligaments can increase the risk of subsequent OA [2, 21]. Our findings align with those in another U.K.-based cohort in which the onset of knee pain was significantly associated with baseline knee injury (OR 1.59, 95% CI 1.17–2.17) over a 3-year period [22]. Knee malalignment is another recognised biomechanical risk factor for the development and progression of KOA, and we previously reported that self-reported constitutional varus malalignment associates with increased incident knee pain (OR 2.82, 95% CI 1.57–5.06) over a 10-year period [17]. A varus alignment creates a knee adduction moment which increases joint loading, particularly on the medial tibiofemoral compartment [23]. In the present study, self-reported varus or valgus alignment had an OR of 3.93 (95% CI 2.14–6.57) for predicting knee pain at 12-year follow-up. Whilst Sharma and colleagues [23] relied on x-ray images for analysis of alignment and load bearing axes, our method uses a simple and cost-effective self-reported measure which has been validated previously [17] and which can be included as part of routine clinical assessment.

Pain elsewhere was a significant risk factor for development of knee pain in our cohort, with an OR of 2.49 (95% CI 1.83–3.30). This is in keeping with longitudinal studies [22] and prevalence literature [24, 25] which have particularly focused on regional body pain at the hip and back. The same definition of pain elsewhere (presence of hip pain and back pain) was used in both the Nottingham and OAI cohorts. It is possible that a proportion of self-reported knee pain could be referred pain from the hips or spine rather than pain originating at the knee. However, simple enquiry concerning other features of the pain (e.g., localised or diffuse, associated with sensory disturbance, improved by rubbing, exacerbated by use or straining) together with a basic musculoskeletal examination should permit ready distinction in primary care without the need for any investigations.

There are several caveats to this study. Firstly, the model performed poorly in the OAI population in the United States. This may be because OAI selected individuals with a higher risk for KOA [26]. The OAI consists of participants with either established KOA or significant risk factors for the development of KOA to help identify and characterise the disease from onset to joint replacement. Incidentally, 853 OAI participants included in this study had available KL grading, and their data (see Additional file 1: Appendix S1) demonstrated that 317 participants (37.15%) showed definite signs of osteoarthritis (joint space narrowing and osteophyte formation) with KL ≥2, whereas 512 participants (60%) had some signs of osteoarthritis (KL ≥1). By contrast, the Nottingham participants were derived randomly from the community and were at much lower risk of knee pain. There were statistically significant differences in key risk factors at baseline between the two populations, such as age, BMI and injury (Table 1). As a result, the model lost its power to differentiate the cases in hospitals, because those are more likely to be severe cases within a narrower band of the disease spectrum. It suggests that the developed model may be more useful for a community setting, such as in primary care. An alternative to this approach would be to develop the model for OAI and verify that it could not predict the Nottingham population, which would strengthen the obvious discrepancy between these two population sources. Secondly, although we successfully validated the model in the community, this is only an internal validation. We still do not know whether this community-based knee pain prediction model is useful for other community populations, such as a European or U.S. population sample. Therefore, further validation is required. Thirdly, the Nottingham knee pain cohort is a retrospective cohort with only two time points for dichotomous outcomes (knee pain-positive and knee pain-negative). Therefore, it was not possible to apply a time-to-event or survival analysis to maximise information on knee pain incidence. There is an inherent bias to retrospective study designs, such as the inability to accurately recall exposures prior to the study owing to selective preconceptions about the association between risk factors and the knee pain (outcome). Furthermore, this paper is based purely on knee pain outcomes as opposed to structural change from KOA (i.e., evidence of radiographic OA), owing to the lack of knee x-rays available for all 1822 participants in the Nottingham cohort. The prediction can be limited only to knee pain, not to KOA.

## Conclusions

A novel model for predicting knee pain in the general population has been developed. To our knowledge, this is the first knee pain prediction paper based on a large community sample. The preliminary validation demonstrated that the model has high specificity, includes risk factors that can be identified easily in a clinical setting, and is therefore very useful for knee pain prediction in primary care but not in secondary care.

## Additional files

Additional file 1:
**Appendix S1.** KL grading of OAI participants. (DOCX 15 kb)

## Abbreviations

BMI: Body mass index
2D:4D: Second digit/fourth digit ratio
HLS: Hosmer-Lemeshow χ^2^ statistic
KL: Kellgren and Lawrence
KOA: Knee osteoarthritis
OA: Osteoarthritis
OAI: Osteoarthritis Initiative

## References

[1] Peat G, McCarney R, Croft P (2001). Knee pain and osteoarthritis in older adults: a review of community burden and current use of primary health care. Ann Rheum Dis.

[2] Hunter DJ, Felson DT (2006). Osteoarthritis. BMJ.

[3] Hootman JM, Helmick CG (2006). Projections of US prevalence of arthritis and associated activity limitations. Arthritis Rheum.

[4] Arthritis Care (2012). OA nation 2012.

[5] Lawrence RC, Felson DT, Helmick CG, Arnold LM, Choi H, Deyo RA (2008). Estimates of the prevalence of arthritis and other rheumatic conditions in the United States: part II. Arthritis Rheum.

[6] Zhang W, McWilliams DF, Ingham SL, Doherty SA, Muthuri S, Muir KR (2011). Nottingham knee osteoarthritis risk prediction models. Ann Rheum Dis.

[7] Ho-Pham LT, Lai TQ, Mai LD, Doan MC, Pham HN, Nguyen TV, Milanese S (2014). Prevalence of radiographic osteoarthritis of the knee and its relationship to self-reported pain. PLoS ONE.

[8] Link TM, Steinbach LS, Ghosh S, Ries M, Lu Y, Lane N (2003). Osteoarthritis: MR imaging findings in different stages of disease and correlation with clinical findings. Radiology.

[9] Hadler NM (1992). Knee pain is the malady—not osteoarthritis. Ann Intern Med.

[10] Hannan MT, Felson DT, Pincus T (2000). Analysis of the discordance between radiographic changes and knee pain in osteoarthritis of the knee. J Rheumatol.

[11] Dawson J, Linsell L, Zondervan K, Rose P, Carr A, Randall T (2005). Impact of persistent hip or knee pain on overall health status in elderly people: a longitudinal population study. Arthritis Rheum.

[12] Brooks PM (2006). The burden of musculoskeletal disease--a global perspective. Clin Rheumatol.

[13] Kerkhof HJ, Bierma-Zeinstra S, Hofman B, Rivadeneira F, Uitterlinden A, Janssens C (2013). OP0130 Prediction model for knee osteoarthritis including clinical, genetic and biochemical risk factors. Ann Rheum Dis.

[14] Ingham SL, Zhang W, Doherty SA, McWilliams DF, Muir KR, Doherty M (2011). Incident knee pain in the Nottingham community: a 12-year retrospective cohort study. Osteoarthritis Cartilage.

[15] Kleinbaum DG, Kupper LL, Morgenstern H (1982). Epidemiologic research principles and quantitative methods.

[16] Zhang W, Robertson J, Doherty S, Liu JJ, Maciewicz RA, Muir KR, Doherty M (2008). Index to ring finger length ratio and the risk of osteoarthritis. Arthritis & Rheumatism.

[17] Ingham SL, Moody A, Abhishek A, Doherty SA, Zhang W, Doherty M (2010). Development and validation of self-reported line drawings for assessment of knee malalignment and foot rotation: a cross-sectional comparative study. BMC Med Res Methodol.

[18] Hosmer DW, Lemeshow S. Multiple Logistic Regression. Applied Logistic Regression: John Wiley & Sons, Inc; 2005.

[19] Bland JM, Altman DG. Survival probabilities (the Kaplan-Meier method). BMJ. 1998;317(7172):1572-80. 10.1136/bmj.317.7172.1572 PMC1114388 9836663

[20] O'Hagan, A. Probability: methods and measurement. London: Chapman and Hall; 1988.

[21] Lohmander LS, Ostenberg A, Englund M, Roos H. High prevalence of knee osteoarthritis, pain, and functional limitations in female soccer players twelve years after anterior cruciate ligament injury. Arthritis Rheum. 2004;50(10):3145-52. 10.1002/art.20589 15476248

[22] Jinks C, Jordan KP, Blagojevic M, Croft P. Predictors of onset and progression of knee pain in adults living in the community. A prospective study. Rheumatology. 2008;47(3):368-74. 10.1093/rheumatology/kem374 18263594

[23] Sharma L, Song J, Felson DT, Cahue S, Shamiyeh E, Dunlop DD (2001). The role of knee alignment in disease progression and functional decline in knee osteoarthritis. JAMA.

[24] Croft P, Jordan K, Jinks C (2005). "Pain elsewhere" and the impact of knee pain in older people. Arthritis Rheum.

[25] Cecchi F, Mannoni A, Molino-Lova R, Ceppatelli S, Benvenuti E, Bandinelli S, Lauretani F, Macchi C, Ferrucci L (2008). Epidemiology of hip and knee pain in a community based sample of Italian persons aged 65 and older. Osteoarthritis Cartilage.

[26] Nevitt MC, Felson DT, Lester G. The Osteoarthritis Initiative: a Knee Health Study. 2006. Available online: https://oai.epi-ucsf.org/datarelease/docs/StudyDesignProtocol.pdf.

