# Supplementary material for: Risk prediction model for knee pain in the Nottingham community: a Bayesian modelling approach
Source: Arthritis Res Ther. 2017 Mar 20;19:59. doi: 10.1186/s13075-017-1272-6 (PMC5359844; doi:10.1186/s13075-017-1272-6)
Supplement: Additional file 1: — Appendix S1. KL grading of OAI participants. (DOCX 15 kb) [file 13075_2017_1272_MOESM1_ESM.docx]

Appendix 1.

| OAI KL Grading | N (of 853) | Proportion (%) |
| --- | --- | --- |
| Grade 0 | 341 | 39.97 |
| Grade 1 | 195 | 22.86 |
| Grade 2 | 229 | 26.84 |
| Grade 3 | 79 | 9.26 |
| Grade 4 | 9 | 1.05 |

Table showing OAI KL grading in OAI participants (n=2 missing)
